# Unveiling the hidden role of extracellular vesicles in brain metastases: a comprehensive review

**DOI:** 10.3389/fimmu.2024.1388574

**Published:** 2024-04-25

**Authors:** Ji Li, Shuangqing Lu, Feihu Chen, Hui Zhu

**Affiliations:** Department of Radiation Oncology, Shandong Cancer Hospital and Institute, Shandong First Medical University and Shandong Academy of Medical Sciences, Jinan, Shandong, China

**Keywords:** brain metastasis, extracellular vesicles, immune microenvironment, molecular mechanism, immunotherapy

## Abstract

**Background:**

Extracellular vesicles (EVs) are small, transparent vesicles that can be found in various biological fluids and are derived from the amplification of cell membranes. Recent studies have increasingly demonstrated that EVs play a crucial regulatory role in tumorigenesis and development, including the progression of metastatic tumors in distant organs. Brain metastases (BMs) are highly prevalent in patients with lung cancer, breast cancer, and melanoma, and patients often experience serious complications and are often associated with a poor prognosis. The immune microenvironment of brain metastases was different from that of the primary tumor. Nevertheless, the existing review on the role and therapeutic potential of EVs in immune microenvironment of BMs is relatively limited.

**Main body:**

This review provides a comprehensive analysis of the published research literature, summarizing the vital role of EVs in BMs. Studies have demonstrated that EVs participate in the regulation of the BMs immune microenvironment, exemplified by their ability to modify the permeability of the blood-brain barrier, change immune cell infiltration, and activate associated cells for promoting tumor cell survival and proliferation. Furthermore, EVs have the potential to serve as biomarkers for disease surveillance and prediction of BMs.

**Conclusion:**

Overall, EVs play a key role in the regulation of the immune microenvironment of brain metastasis and are expected to make advances in immunotherapy and disease diagnosis. Future studies will help reveal the specific mechanisms of EVs in brain metastases and use them as new therapeutic strategies.

## Introduction

Metastases are typically associated with unfavorable prognoses and represent the primary cause of mortality in cancer patients (1). Brain metastasis (BMs) is a common clinical occurrence, particularly in patients with advanced non-small-cell lung cancer (NSCLC), with BMs developing in up to 40% of cases (2). The majority of patients with brain metastases experience significant changes in nervous system function, adversely impacting their quality of life. However, due to the limited treatment options, the prognosis remains unfavorable. As a result, brain metastases significantly impact the survival time and quality of life for patients. Early-stage patients with BMs often face challenges in receiving optimal treatment due to a lack of symptoms. However, there is still a lack of noninvasive and highly accurate tumor biomarkers in the early stages, which could play a significant role in BMs screening. Despite substantial progress in chemotherapy, radiotherapy, and targeted therapy, patients with advanced BMs continue to have a poor prognosis, imposing a substantial burden on families and society. Therefore, there is an urgent need for additional prognostic and risk indicators.

In the current phase of scientific exploration and implementation, researchers are investigating the structure, related technologies, and mechanisms of exosomes. A significant milestone was reached at the end of 2018 when the International Society for EVs issued guidelines to establish standardized nomenclature for EVs. Presently, the majority of techniques used for isolating exosomes result in the isolation of heterogeneous populations of EVs derived from various biogenic sources. For the sake of precision and clarity, we will henceforth refer to these vesicles as “EVs,” which are commonly referred to as “exosomes” in scientific literature. The Minimal Information for Studies of Extracellular Vesicles 2018 (MISEV2018) guidelines specify the use of membrane vesicles derived from small cells (3).

EVs, composed of nanoscale vesicle structures secreted by the majority of cells, comprise three distinct types of vesicles, exosomes (30-150 nm), microvesicles (100-1000 nm), and apoptotic bodies (1000-5000 nm). Cells can secrete exosomes in various states, apoptotic bodies are secreted during apoptosis, and microparticles are released when cells receive external stimuli such as radiation. EVs play a pivotal role in intercellular communication and participate in various physiological functions (4). As an illustration, the cargo of fatty acids carried by tumor EVs and particles (EVPs), specifically palmitic acid, induces the secretion of tumor necrosis factor (TNF) by Kupffer cells. This process creates a pro-inflammatory microenvironment, inhibits fatty acid metabolism and oxidative phosphorylation, and contributes to the formation of fatty liver (5). Moreover, studies have indicated that tumor type-specific proteins present in EVPs can aid in the classification of unknown primary tumors. The protein content of EVPs can serve as a dependable biomarker for the detection and identification of different types of cancer (6). The aforementioned studies demonstrate the significant impact of EVs on the onset and progression of tumors; however, the influence on brain metastases remains uncertain. The presence of the blood-brain barrier represents a fundamental distinction between the brain and other bodily tissues and organs. Owing to the distinctive structure of EVs, they possess the ability to traverse the blood-brain barrier and influence the onset and progression of brain metastases. In recent times, there has been a growing number of articles that elucidate the role of EVs or exosomes in the context of brain metastases. This surge in research output has fueled the expansion of exosome-related investigations in this field.

The objective of this review is to present a comprehensive overview of the recent major advancements in the field, focusing on the role and application of EVs in brain metastases. A comprehensive literature search was conducted using Embase, PubMed, Web of Science, and Clinicaltrials.gov databases to identify relevant publications until November 1, 2023. The screening process for literature pertaining to “Brain metastasis” [Mesh], “Exosomes” [Mesh], and “Extracellular vesicles” adhered to the guidelines established in MISEV 2018.

## Characteristics of extracellular vesicles

### Structure and Biological Origin of Extracellular Vesicles

**Figure 1:**
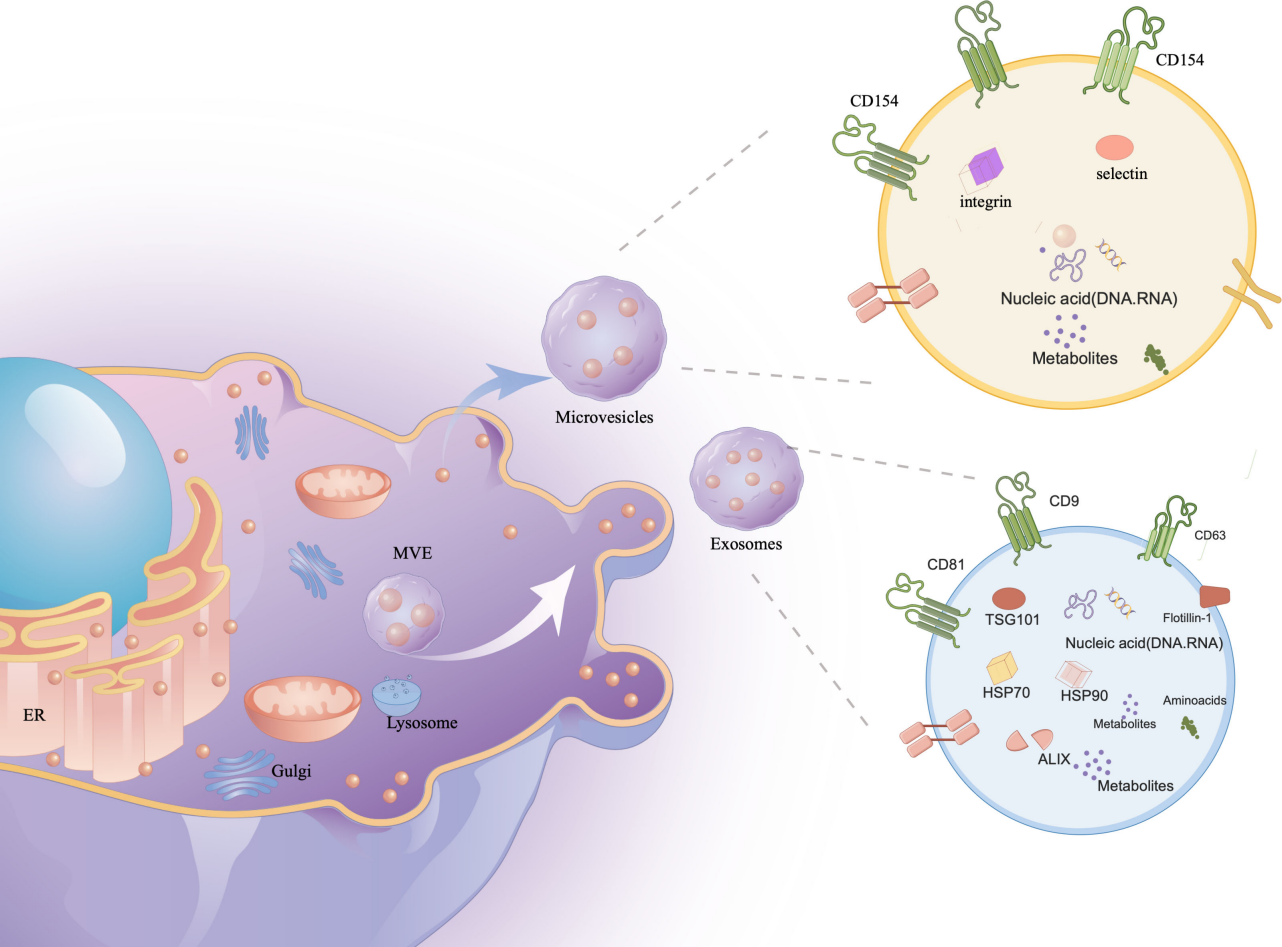
The contents of exosomes. The cell membrane invagination will form endosome, which will then form multivesicular bodies (MVB), which will be secreted to the exocytosomes as exosomes. Exosomes contain a variety of proteins, lipids, DNA, RNA and other important information of the mother cell.

In their study, researchers observed the transformation of sheep reticulocytes into mature red blood cells, during which they release small vesicles containing transferrin metabolites. Initially dismissed as cell debris, further research recognized these vesicles as distinct structures (7). The process begins with the plasma membrane forming early endosomes, which evolve into multivesicular bodies (MVBs) through vesicle formation (8). MVBs are crucial for various cellular functions, including endocytosis, protein sorting, storage, transport, and the release into the extracellular space, either by degradation or fusion with the cell membrane (9). Although the endosomal sorting complex required for transport (ESCRT) has been implicated in exosome formation, evidence suggests ESCRT-independent pathways also play significant roles (10–12). Novel mechanisms, such as one controlled by RAB31, further expand our understanding of exosome biogenesis (**Figure 1**).

Extracellular vesicles (EVs) are categorized into exosomes (30-150 nm), microvesicles (100-1000 nm), and apoptotic bodies (1000-5000 nm) (13, 14), each carrying vital biological materials like proteins, DNA, RNA, enzymes, and lipids (15, 16). Initially mistaken for waste, these vesicles are now recognized for their critical roles in human development and regulation. They influence biomarker levels and act as therapeutic agents and drug delivery systems, especially in autoimmune diseases (17–19).

Exosomes are considered the most applicable vesicles due to their nanometric size and ease of isolation. These single-cell exosomes have a membrane structure composed of bilayers of lipids, measuring 30-150 nm in size and having a density of approximately 1.13-1.21 g/ml (20). Exosomes are produced in most human cells and are widely distributed in various bodily fluids, including blood, urine, cerebrospinal fluid, tears, saliva, milk, ascites, lymph, and amniotic fluid (21). Ectosomes (difference with exosomes), also known as microparticles or microvesicles, derived from plasma membranes, exhibit similar functions to exosomes (22). Apoptotic bodies, observed during apoptosis, are also referred to as apoptotic vesicles. They can be mistakenly identified as other EVs (23). The functionality of EVs largely relies on their complex and diverse cargo. Approximately 76% of this cargo consists of proteins, while 15% comprises mRNA. The remaining components include DNAs, microRNAs (miRNAs), circular RNAs (circRNAs), and long noncoding RNAs (lncRNAs). These components have the potential to significantly alter recipient cells that interact with exosomes (24–38).

In the current state, there is a lack of optimal separation strategies or markers for distinguishing between different sources of EVs. Therefore, it is challenging to propose specific and universally applicable markers for MVB-derived “exosomes” compared to other small EVs. The term “exosomes” refers to EV preparations that have been isolated from larger vesicles, but these preparations are actually mixtures of exosomal and non-exosomal EV particles (29). Following the guidelines of MISEV2018, we have used the term “small extracellular vesicles (sEVs)” to refer to vesicles with diameters of either 200 nm or 100 nm, instead of using the term “exosomes” (4).

### Characterization, storage, and separation of sEVs

Various protocols have been employed for the separation of sEVs (30, 31).Currently, ultracentrifugation is widely used for the isolation of sEVs (32). Classic techniques, including density gradients (33), immunoisolation (34), precipitation (35), and filtration (36), are also utilized. Each method has advantages and disadvantages in terms of recovery, specificity, time, and cost (37, 38). Despite the development of several novel techniques in recent years (39–42), complete isolation of sEVs remains challenging. Therefore, a combination of methods will remain recommended in the future. Identifying sEVs is also challenging. MISEV2018 guidelines recommend providing quantitative descriptions of both the EV source and EV preparation. EV characteristics are routinely assessed by detecting and analyzing protein content. For positive identification, EVs must contain at least one transmembrane/lipid-bound protein (typically CD9, CD63, CD81, and integrin) and one cytosolic protein (typically ALIX, TSG101, syntenin, and HSP70). Additionally, the levels of at least one negative protein, such as albumin, lipoproteins, or ribosomal proteins, must be determined. Furthermore, analysis of functional proteins, including histones, cytochrome C, calnexin, and Grp94 (7), is required for sEVs.

Western blotting is the most commonly used method for detecting proteins on the surface and within a cell. Fluorescent microscopy enables the detection of structures that have been labeled with fluorescent dyes (43). Flow cytometry has limitations in detecting SEVs due to their small size and low amount of surface antigens (44). In recent years, there has been a significant improvement in the affordability and accessibility of mass spectrometry techniques (45). However, when it comes to protein extraction, a large quantity of small extracellular vesicles (sEVs) is required, which can often reduce the overall efficiency of the process (9). To fully characterize the heterogeneity of individual vesicles, it is recommended to employ two complementary techniques, such as transmission electron microscopy or atomic force microscopy (46). These imaging techniques provide valuable insights into the structural characteristics of sEVs, allowing for a better understanding of their composition and behavior. In addition to extracting and characterizing sEVs, proper storage conditions are essential for maintaining the integrity and preserving the characteristics of these vesicles. Currently, there is no general consensus regarding the storage conditions for the original samples from which sEVs are extracted, specifically whether they should be stored at -80°C and used promptly when conducting experiments (47, 48).

## EVs regulate the microenvironment of brain metastases

### EVs affect the pre-metastatic immune microenvironment

Recent studies have demonstrated the crucial role of exosomes in establishing a pre-metastatic immune microenvironment for brain metastasis. Hoshino et al. conducted an analysis on the biodistribution of tumor-secreted exosomes and discovered that integrins (ITGs) fuse with T cells in a tissue-specific manner, thereby facilitating organ-specific colonization and creating a pre-metastatic microenvironment for brain metastasis (49). Moreover, tumor-secreted CEMIP^+^ exosomes are taken up by brain endothelial and microglial cells, resulting in the upregulation of pro-inflammatory cytokines encoded by Ptgs2, Tnf, and Ccl/Cxcl, which promotes brain vascular remodeling and metastasis (50). This suggests that exosomes have the ability to modify the premetastatic immune microenvironment, facilitating tumor metastasis to target organs. Additionally, researchers have discovered that cancer-derived extracellular miR-122 modifies glucose utilization in recipient premetastatic niche cells, leading to the reprogramming of systemic energy metabolism to aid disease progression. Consequently, glucose becomes more readily available to metastatic tumor cells in the brain, allowing for their initial expansion (51). Moreover, miR-19a transferred from astrocyte EVs to metastatic breast cancer cells downregulates PTEN and increases proliferation of the recipient tumor cells (52). It has also been observed that the expression of serum exo-AnxA2 is elevated in African-American (AA) women with triple-negative breast cancer (TNBC), promoting angiogenesis (53). EVs derived from breast tumors can interact with Toll-like receptor 2 (TLR2) on macrophages, stimulating the nuclear factor kappa-light-chain-enhancer of activated B cells (NF-κB) signaling pathway and increasing the production of pro-inflammatory cytokines such as IL-6 and tumor necrosis factor alpha (TNF-α) (54). Additionally, these EVs released from drug-resistant MCF7 breast cancer cells stimulate IL-6 expression and decrease macrophage chemotaxis (55). Tumor-derived EVs containing tumor necrosis factor-related apoptosis inducing ligand (TRAIL) induce apoptosis *in vitro* (using the oligodendroglioma G26/24 cell line) (56). Furthermore, in breast cancer, loss of XIST activates MSN-c-Met and reprograms microglia through exosomal miR-503, thus promoting brain metastasis (57). miR-210 has been reported as a pro-angiogenic miRNA in normal adult mouse brain (58) and is overexpressed in cells that specifically metastasized to the brain (59). The possibility of EV miR-210 participating in brain metastasis and angiogenesis of breast cancer needs further study.

Researchers have demonstrated that EVs derived from melanoma, particularly exosomes, activate pro-inflammatory signaling in both lung fibroblasts and astrocytes (60). This exosome-mediated pro-inflammatory reprogramming plays a crucial functional role in the recruitment of immune cells by activated fibroblasts and astrocytes (61). Additionally, blasts from B cell precursor (BCP)-acute lymphoblastic leukemia (ALL) release multiple cytokines and exosomes containing IL-15. These exosomes bind to and are internalized by astrocytes and brain vessel endothelial cells. Consequently, astrocytes produce VEGF-AA, which disrupts the integrity of the blood-brain barrier (BBB) (62).

**Figure 2:**
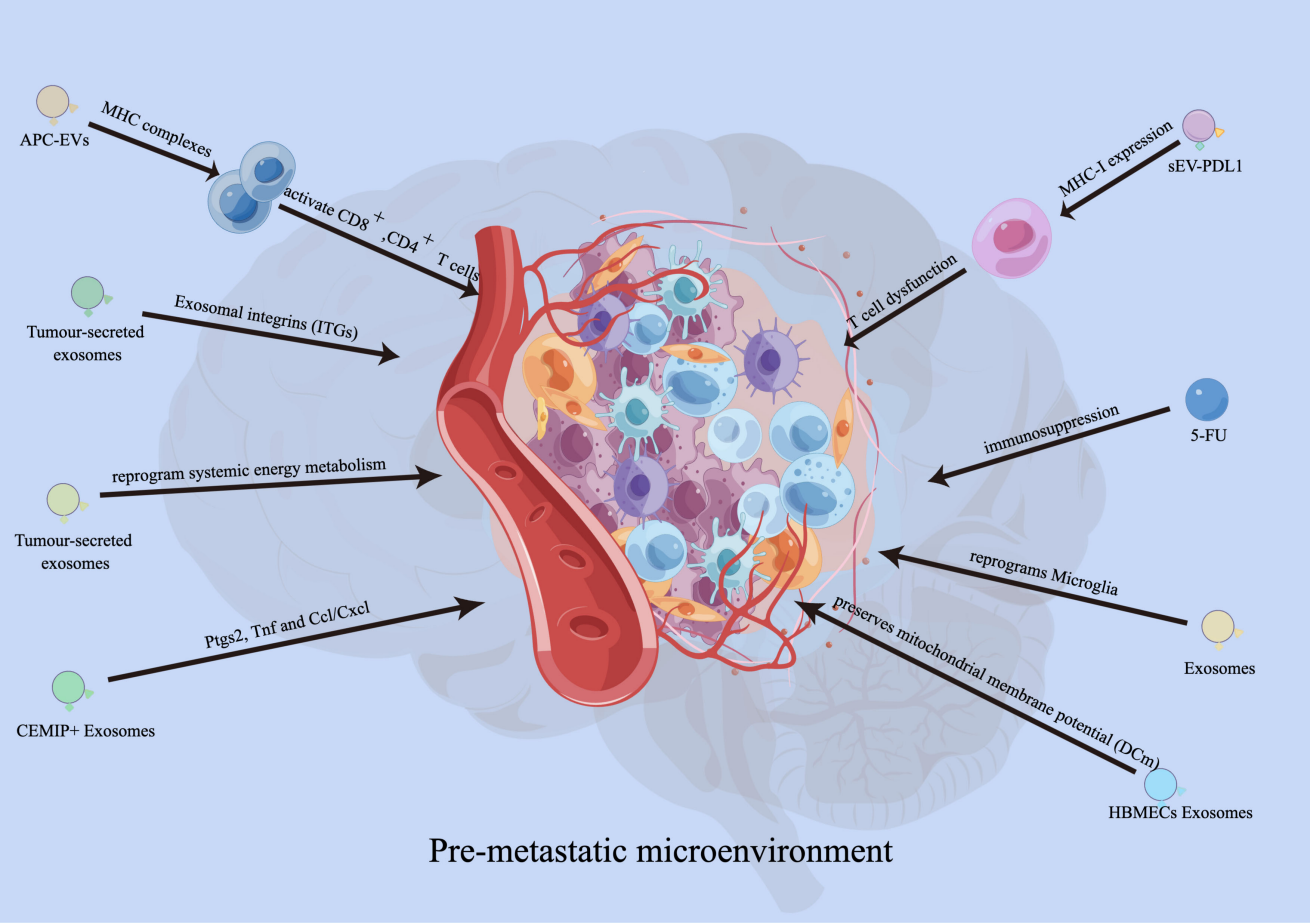
Functions of EVs in developing pre-metastatic microenvironment. EVs derived from antigen presenting cells (APCs) can also activate CD8^+^and CD4^+^ T cells. Exosomes carrying PD-L1 promoted tumor growth and reduced the number of T cells in the spleen and lymph nodes in mouse experiments. Integrins (ITGs) fuse with target cells in a tissue-specific manner to direct organ-specific colonization. Tumour-secreted CEMIP^+^ exosomes uptaked by brain endothelial and microglial cells, upregulating the pro-inflammatory cytokines encoded by Ptgs2, Tnf and Ccl/Cxcl, promote brain vascular remodelling and metastasis. Human brain microvascular endothelial cells (HBMECs)-derived exosomes induce the elevated S100A16 in SCLC brain metastasis, and the S100A16-mediated protective effect is related to the up-regulation of prohibitin (PHB)-1.

Exosomes derived from human brain microvascular endothelial cells (HBMECs) induce an increase in S100A16 levels in SCLC brain metastasis. The protective effect mediated by S100A16 is related to the up-regulation of prohibitin (PHB)-1, a protein found in the mitochondria’s inner membrane. PHB-1 helps preserve mitochondrial membrane potential (DCm) and supports the survival of SCLC cells in the brain (63). Zhang and colleagues have recently shown that miR-19a from astrocytes downregulates PTEN expression in cancer cells. This downregulation leads to increased secretion of CCL2 and recruitment of myeloid cells, ultimately promoting brain metastasis (52). Furthermore, the transfer of exosomal cargo induces transcriptomic changes that enhance the inflammatory phenotype of stromal cells, similar to that of cancer-associated fibroblasts (CAFs) (64). Overall, extracellular vesicle-derived miRNA, proteins, and genes contribute to the creation of a pre-metastatic microenvironment and promote tumor metastasis by influencing the phenotype of intracranial immune cells while improving the metastatic ability of tumor cells (**Figure 2**).

### EVs affect blood-brain barrier permeability

The central nervous system (CNS) is tightly regulated by the blood-brain barrier (BBB) and the neurovascular unit (NVU), composed of endothelial cells (ECs), pericytes, and astrocytic endfeet, to ensure normal brain function (65). The primary event in brain metastasis is the infiltration of cancer cells through the blood-brain barrier (BBB). Researchers have demonstrated that exosomes can cross the BBB and transport cargo, bypassing the mononuclear phagocyte system (MPS) (66). Tumor-derived EVs can breach the intact BBB *in vivo* by utilizing transcytosis as the underlying mechanism. Researchers have also identified and characterized the mechanism by which tumor-derived EVs overcome the low physiological rate of transcytosis in the BBB. This is achieved by reducing brain endothelial expression of rab7 and enhancing transport efficiency (67). A study demonstrated that TGF-β1-mediated exosomal lnc-MMP2-2 derived from non-small cell lung cancer (NSCLC) increases BBB permeability and facilitates brain metastasis of NSCLC (68). In addition, lncRNA GS1-600G8.5 was found to be significantly upregulated in breast cancer cells with a high propensity for brain metastasis, in contrast to exosomes derived from poorly metastatic breast cancer cells. Disruption of the BBB by exosomal lncRNA GS1-600G8.5 promoted the passage of breast cancer cells, potentially through the targeting of tight junction proteins. These studies have provided a novel understanding of the role of exosomal lncRNAs in cancer brain metastasis (69) (**Table 1**). Evs derived from MDA-MB-231 breast cancer cells reduce the expression of the tightly connected molecule ZO-1 by loading miR-105 (70). Another study on BMS breast cancer cells also confirmed the effect of EV miR-181c on the localization of actin filaments, resulting in increased brain endothelial permeability (71). These may be the potential mechanisms by which tumor-derived EVs affects the integrity of the blood-brain barrier. The findings indicate that EVs can compromise the integrity of the blood-brain barrier and facilitate the formation of brain metastases.

**Table 1:** Role of sEVs in brain metastasis.

Type	Contents	Donor cells	Recipient cells	Function	Ref.
*Protein*	ITGs	Cancer cells	T cells	establish pre-metastatic microenvironment	(49)
*Protein*	CEMIP	Cancer cells	brain endothelial and microglial cells	promote brain vascular remodelling and metastasis	(50)
*Protein*	TRAIL	G26/24 cell line	Astrocyte	induce astrocyte apoptosis *in vitro*	(56)
*Protein*	IL-15	BCP-ALL	astrocytes, brain vessel endothelial cells	disrupts the integrity of the blood-brain barrier (BBB)	(58)
*Protein*	S100A16	HBMECs	Cancer cells	supports the survival of SCLC cells in brain	(59)
*miRNA*	miR-19a	Astrocyte	Cancer cells	enhanced the proliferation of the recipient tumour cells	(60)
*miRNA*	miR-122	Cancer cells	niche cells	reprogram systemic energy metabolism to facilitate disease progression	(51)
*miRNA*	miR-503	Cancer cells	Microglia	promote Brain Metastasis	(53)
*lncRNA*	lnc-MMP2-2	Cancer cells	brain vessel endothelial cells	increases BBB permeability	(66)
*lncRNA*	lncRNA GS1-600G8.5	Cancer cells	brain vessel endothelial cells	disrupted the BBB	(67)
*miRNA*	miR-210	Cancer cells	brain vessel endothelial cells	promote brain vascular remodelling and metastasis	(58)
*miRNA*	miR-105	Cancer cells	brain vessel endothelial cells	disrupted the BBB	(70)
*miRNA*	miR-181c	Cancer cells	brain vessel endothelial cells	disrupted the BBB	(71)
*Protein*	IL-6	Cancer cells	Macrophage	promote Brain Metastasis	(55)

### EVs affect the survival and proliferation of tumor cells

Tumor suppressor genes, such as PTEN, encode the phosphatase PTEN. Expression of PTEN in metastatic tumor cells is hindered by the presence of EV-miR-19 released from astrocytes. Moreover, tumor cells lacking PTEN engage in the recruitment of IBA1^+^ myeloid cells to enhance proliferation. Consequently, the recruited myeloid cells secrete NF-kb and CCL2, thereby inhibiting their own apoptosis and promoting the occurrence of brain metastasis (72). The underlying phenomenon can be illustrated as follows. The miRNA derived from stromal cells within the tumor microenvironment stimulates cell proliferation while concurrently inhibiting cell apoptosis, thus promoting tumor growth.

Tumor-derived exosomes have the potential to enhance tumor cell proliferation. A recent study observed an increase in the expression of miR-503, a negative regulator of the X-inactive specific transcript, in breast cancer patients with brain metastasis. Subsequently, the upregulation of miR-503 in microglia promotes the transformation from an M1 to an M2 phenotype (73). The acquisition of an M2 phenotype by microglia facilitates an immunosuppressive tumor environment by impeding T cell proliferation. These findings confirm that tumor-derived exosomes have the ability to modulate immune cell function, promoting immune evasion mechanisms and creating a conducive environment for tumor cell proliferation (74). Exosomes derived from antigen presenting cells (APCs), in addition to containing MHC complexes, have the ability to directly or indirectly activate CD8^+^ T cells and CD4^+^ T cells (75). Dendritic cell-derived exosomes are capable of inducing a pro-inflammatory cytokine profile, while exosomes derived from tumor cells typically induce a pro-tumorigenic immune profile. Remarkably, exosomes carrying PD-L1, identical in structure to the surface of tumor cells, are capable of binding to T cells (76). An experiment conducted with CD8^+^ T cells demonstrated that PD-L1-incorporated exosomes inhibit their proliferation. In mouse experiments, exosomes carrying PD-L1 were found to promote tumor growth and decrease the number of T cells in the spleen and lymph nodes (76). During the early stages of apoptosis, decline in mitochondrial membrane potential and increase in radical production can be observed. Exosomes play a significant role in preventing the decline in mitochondrial membrane potential during the early stages of apoptosis through the actions of PHB, a protein found in the mitochondrial inner membrane (77). These findings suggest that exosomes have the ability to regulate tumor cell stability and promote their proliferation by modulating mitochondrial membrane potential. Overall, EVs, particularly exosomes, can significantly impact the survival and proliferation of tumor cells through their influence on mitochondrial function.

## Application potential of extracellular vesicles in disease monitoring and prediction of BMs

### EVs as potential tumor markers for brain metastasis

Pathologists commonly perform tissue biopsies for cancer diagnosis and treatment monitoring purposes. In contrast, liquid biopsies offer minimal invasiveness, the potential for serial testing, and the ability to detect cancer at an earlier, more treatable stage. With rising expectations for liquid biopsy technologies, exosomes are emerging as a valuable resource for early cancer detection.

It has been demonstrated in previous studies that cells release exosomes, abundant in blood, cerebrospinal fluid (CSF), and urine. Currently, the detection method is becoming more specialized and sensitive. *In vitro* experiments showed higher expression levels of these exosome miRNAs in CRC cell lines compared to non-tumor cells (78). Patients with gastric cancer (GC) exhibited a significant increase in plasma LINC00152 levels compared to healthy controls (79). Exo-miRNAs can serve as biomarkers for early-stage prognosis of brain metastases. Skog et al. (80) previously reported the isolation of serum-derived EVs from patients with brain tumors and the detection of specific genetic alterations in the EGFR gene. Cerebrospinal fluid (CSF) has been found to be a suitable biofluid for analyzing the macromolecular contents of EVs in previous EV studies. Chen et al. demonstrated that mutant IDH1 G395A can be detected in CSF EVs with a sensitivity of 63% and a specificity of 100% (81). Figueroa et al. (2019) reported that, in comparison to the gold standard qPCR method used for detecting the EGFRvIII transcript in brain tumor tissue, extracellular vesicular RNA analysis allows for the detection of the oncogene EGFRvIII with a sensitivity of 60% and a specificity exceeding 98% (82). Manda et al. conducted similar studies on plasma, which yielded an 80% sensitivity and a 79% specificity (83). Akers et al. demonstrated that miR-21 (84) and miRNA signature (85) from CSF EVs can differentiate between tumor and non-tumor disease states.

### EVs as new targets for brain metastasis therapy

Exosomes are believed to play a role in the development, metastasis, and cell proliferation of brain metastasis. Directly targeting the proteins and nucleic acids within exosomes has the potential to suppress cancer development. This research is expected to generate novel insights for the diagnosis and treatment of brain metastasis. Cell surface molecules, such as ANXA2 (86) and miRMA (87), are considered potential targets for brain metastasis therapy. PD-L1 (Programmed Cell Death-Ligand 1) is expressed in EVs secreted by tumors and can metastasize to other cells. Binding of PD-L1 with PD-1 activates the PD-1/PD-L1 pathway, resulting in diminished anti-tumor activity of T cells and induction of T-cell apoptosis. This pathway ultimately facilitates immune escape by tumor cells (76, 88). Reducing the expression of Rab27a and using an exosome inhibitor (GW4869) not only impacted exosome production but also inhibited the release of PD-L1 from exosomes (89). Therefore, these findings have the potential to unleash powerful anticancer effects. This represents a significant stride towards precision medicine and personalized treatment for brain metastasis.

### EVs as new drug delivery systems for brain metastasis therapy

In patients with central nervous system (CNS) metastases, the efficacy of standard chemotherapies or targeted agents is constrained by the limited penetration of antineoplastic agents across the blood-brain barrier (90). Exosomes serve as a promising drug delivery system to overcome the challenge of chemotherapeutic drugs crossing the blood-brain barrier. Using exosomes as drug delivery systems offers several advantages, such as low toxicity, membrane-like structure, flexibility, high drug carrying capacity, passive targeting, good biocompatibility, as well as improved drug bioavailability and sustained release (91, 92). The loading methods of drugs into exosomes can be categorized into two types: endogenous and exogenous approaches (93). In endogenous loading, parent cells undergo genetic modification to express specific proteins or nucleic acids to be packaged in the released vesicles. Alternatively, drugs can be loaded exogenously by incorporating them into exosomes derived from cell culture media or body fluids, such as urine, blood, saliva, or breast milk. Various vesicular systems, including niosomes, proniosomes, ethosomes, ufasomes, pharmacosomes, transferosomes, and phytosomes, have been extensively investigated due to their capability to deliver drugs to the target site while minimizing toxicity to healthy tissues (94).

The study examined zebrafish embryos to assess the effectiveness of exosomes in delivering anticancer drugs to the brain *in vivo* (95). Additionally, experiments with mice demonstrated the ability of exosomes to transport siRNA across the blood-brain barrier and into the brain (96). In an initial study, the researchers investigated whether naturally brain-targeted exosomes, derived from brain endothelial cells, could transport siRNA specific to the tumor marker vascular endothelial growth factor (VEGF) across the blood-brain barrier (BBB) in both *in vitro* and *in vivo* settings. The researchers successfully suppressed zebrafish tumors that were xenografted with VEGF through the delivery of VEGF using exosomes, and subsequently achieved tumor knockdown in zebrafish utilizing the delivered VEGF. These findings provide support for the potential use of natural exosome vesicles in targeted delivery of siRNA to the brain for the treatment of brain diseases (97). In another study, it was demonstrated that exosomes, namely Exo-cur which encapsulated curcumin, and Exo-JSI124 which inhibited signal transducer and activator of transcription 3 (Stat3), could be noninvasively delivered to microglia cells through intranasal administration. The study further revealed the preventive effect of intranasally administered Exo-cur or Exo-JSI124 on LPS-induced brain inflammation in mice (98). A similar study reported that niosomes incorporating folic acid were taken up by rat brain models with an uptake rate of approximately 48.15% (99). This suggests that exosome is a novel treatment stratege for brain tumors and metastases.

## The practical application of EVs in the treatment of brain metastases

sEVs hold immense potential in the field of cancer immunotherapy (49). Immunotherapy-related clinical trials have gradually emerged as a result of the development of PD-1/PD-L1 research for MSI-H and dMMR patients. Recent research in tumor immunotherapy has primarily focused on inhibitors of the programmed death receptor protein 1 (PD-1) and programmed death-ligand 1 (PD-L1) to enhance immunity and overcome immune suppression by activating and promoting immune cells (80). Previous studies have predominantly explored the role of soluble PD-L1, with limited research on sEV-PD-L1. sEVs, with their secretory properties, can harbor inhibitors and killers of T cells within the local tumor microenvironment. Moreover, they possess the ability to migrate to distant sites, potentially facilitating tumor immune evasion (100). According to Fan et al. (101), the stability of sEV-PDL1 and its impact on T cell function can serve as indicators of a patient’s immune status and long-term prognosis. Furthermore, a 2020 study by Zhang et al. (102) revealed that after two or more cycles of chemotherapy, 5-FU can upregulate the expression of sEV-PD-L1, leading to immunosuppression.

## Potential advantages and challenges of EVs as a treatment strategy for BMs

Since the beginning of this decade, EVs have attracted considerable attention. A new perspective on brain metastasis prevention and treatment is presented by this research that expands our understanding of the functions of small extracellular vesicles (sEVs) and the mechanism of development of tumors. In general, BM metastasis-related sEVs are characterized by complex cargo as the primary mechanisms of action. Furthermore, many unknown mechanisms and molecules, as well as communication between BM cells and the tumor microenvironment, contribute to the overall complexity and uncertainty. With the deeper investigation of exosomes, exosomes are expected to become a new target for cancer treatment and potential tool for early diagnosis of brain metastases.

According to these results, exosome-carried molecules can serve as biomarkers for the detection of diseases in its early stages. On the other hand, exosomes show a relatively high stability. Tissue-specific protein in exosome is found to be stable when stored at -80°C or colder, probably for a extended periods of time. Moreover, EVs can be obtained from autologous dissected primary tumor cells in clinical applications, making them biocompatible and safe options for personalized cancer therapy.

Opportunities and challenges coexist, of course. It is unknown which molecules play a dominant role in the comprehensive effect as well as the mechanisms that underlie the complex cargo. Several large-scale and multicenter studies have not been conducted due to immaturity of technology, diversity of detection methods and results, and high detection costs. A further challenge to the universality in existing research may be the genetic differences between East and West, between countries, and even between regions. Thus, scientific findings must be validated before being applied to clinical practice, a collaborative effort between clinicians, pharmacists, and other professional scientists is urgently needed to evaluate the safety, effectiveness, and stability of sEVs. Regardless, it is foreseeable that in the near future, sEVs will be an important tool for accurate early diagnosis and personalized and efficient treatment of cancers due to their unique biological characteristics, offering infinite power to overcome cancers.

### Application potential of nanoscale biomembrane vesicles for brain metastasis therapy

Nanoparticles in nano drugs are commonly categorized as vesicular, lipid-based, or polymeric. Several vesicular systems, such as niosomes, proniosomes, ethosomes, ufasomes, pharmacosomes, transferosomes, and phytosomes, have been extensively studied for their potential to deliver drugs to target sites while minimizing damage to healthy tissues. Consequently, nanoscale biomembrane vesicles show promising potential for treating brain metastases.

### Liposomes

Liposomes are vesicles composed of a lipid bilayer that encapsulates an aqueous core, offer a promising mechanism for drug delivery due to their biocompatibility as nanovesicles and their ability to protect encapsulated drug molecules from degradation (103, 104). Additionally, liposomal preparations have advantages over other nanocarriers and are considered the gold standard in nanomedicine, with numerous clinically approved liposomal products available for various diseases (105). However, their application in the treatment of brain metastases is rarely reported. Conventional liposomes have limitations such as their inability to interact with high-density lipoproteins (HDL) and low-density lipoproteins (LDL) (106). These problems can be overcome by using stealth liposomes, which are modified with biocompatible, inert, and hydrophilic polymers such as polyethylene glycol (PEG), poly(2-ethyl-2-oxazoline) (PEOZ), polyacrylamide (PAA), polyvinyl pyrrolidone (PVP), and poly-2-methyl-2-oxazoline (PMOZ) (107). These liposomal modifications have been extensively studied for the treatment of autoimmune diseases like rheumatoid arthritis (RA) and psoriasis. Recently, liposomal-based vesicular drug delivery systems that can cross the blood-brain barrier (BBB) and target injured vasculature sites in inflammatory tissues have emerged as potential candidates for treating multiple sclerosis (MS) (108). This provides a strong theoretical basis for exploring the application of this drug delivery system in the treatment of brain metastases.

### Niosomes

**Figure 3:**
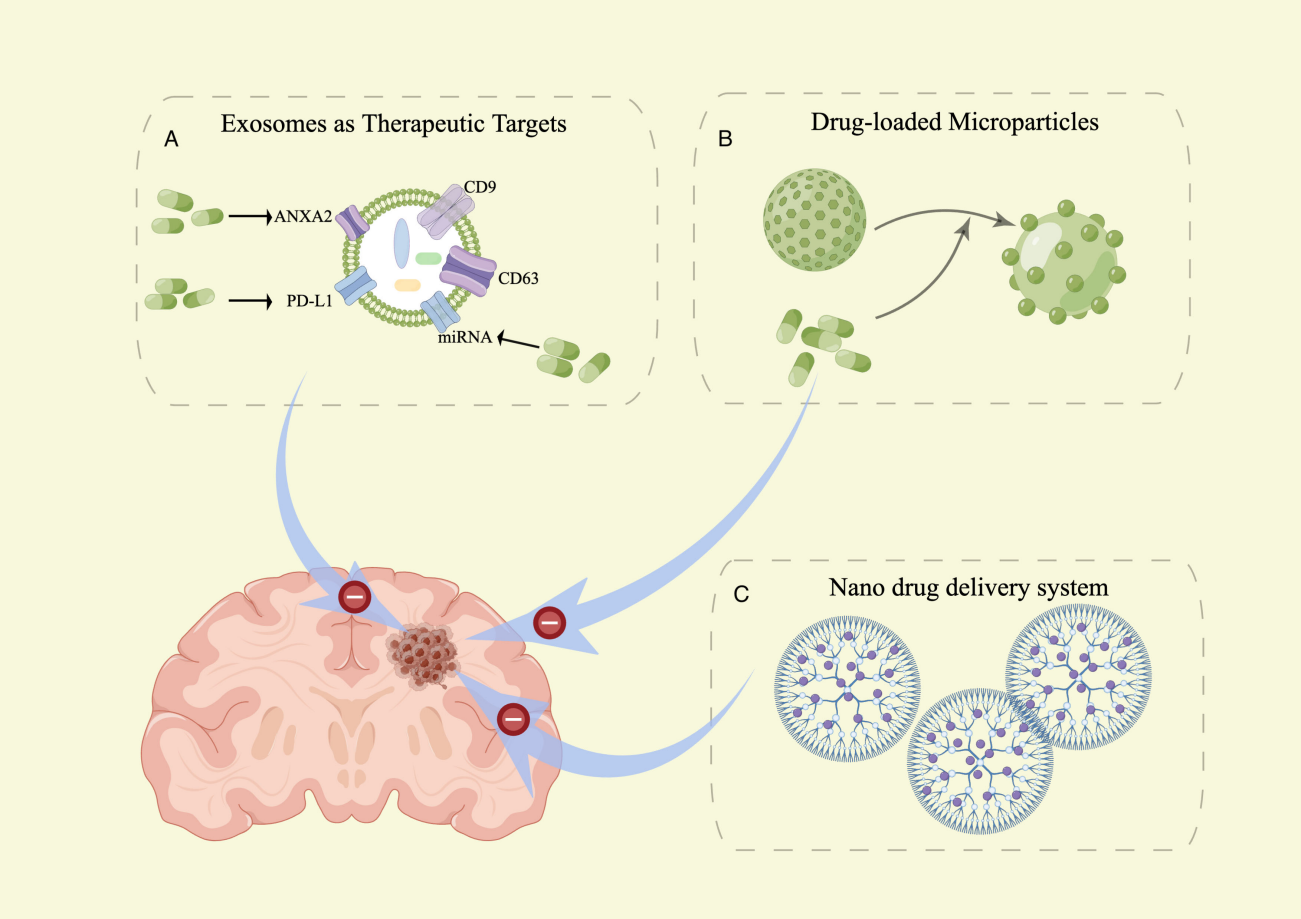
sEVs as tools and targets in brain metastasis therapy. **(A)** sEVs as a new target for brain metastasis therapy. **(B)** sEVs as a new drug delivery system for brain metastasis therapy. **(C)** Article-meta Nanoscale biomembrane vesicles Applications.

Niosomes are a type of vesicular nanocarrier that has revolutionized drug delivery in recent years due to their numerous advantages (109). Niosomes can have different sizes and structures, small unilamellar vesicles (SUVs) are niosomes ranging from 10 to 100 nm in size, while large unilamellar vesicles (LUVs) are niosomes ranging from 100 to 3000 nm in size (110). Studies have demonstrated that the unique surface chemistry of niosomes enables them to cross the blood-brain barrier (BBB) and offers the potential for active targeting through surface modification with ligands (111). Numerous studies have shown that niosomes can cross the blood-brain barrier, offering relief from the symptoms of various autoimmune diseases that impact brain function (112). A similar study reported that niosome-incorporated folic acid was taken up by rat models’ brains at an uptake rate of approximately 48.15% (113) (**Figure 3**). Inflamed areas contain a higher concentration of macrophages, which are immune-related cells responsible for triggering inflammation, making them a primary target for drug development (114). Researchers have shown that targeting macrophages with biocompatible nanovesicular systems can greatly enhance the treatment of autoimmune diseases such as rheumatoid arthritis (RA) (115). Niosomes also hold promise for the treatment of brain metastases.

## Conclusions

EVs serve as crucial intercellular communication mediators with potential benefits and obstacles in managing brain metastases (BMs). EVs transport metastatic factors, miRNA, and proteins, facilitating the migration, colonization, and regulation of cancer cells in brain tissue. Moreover, EVs hold promise as biomarkers for monitoring and predicting the progression and prognosis of brain metastases. However, there are ongoing challenges in addressing the preparation, purification, stability, persistence, safety, and biodistribution of EVs as a therapeutic strategy. Hence, future research should prioritize optimizing EVs’ preparation techniques, developing drug delivery systems, and conducting clinical experiments to enhance their therapeutic potential for brain metastases. Meanwhile, there are more clinical studies targeting primary brain tumors, and there is a lack of the application of EVs as diagnostic and therapeutic drugs for metastatic brain tumors, indicating a limited amount of research on the mechanisms of EVs in metastatic brain tumors. Understanding the mechanism of action of EVs is crucial in guiding the development of effective clinical applications based on EVs.

In conclusion, extracellular vesicles play a significant regulatory role in managing lung cancer brain metastases and have potential value in therapeutic and diagnostic applications. Future studies should prioritize enhancing mechanistic research and conducting clinical trials to promote the practical utilization of extracellular vesicles as a treatment strategy and overcome associated challenges. These efforts will offer brain metastases patients personalized and innovative treatment alternatives, ultimately enhancing their quality of life and prognosis.

## Author contributions

JL: Software, Writing – original draft. SL: Supervision, Writing – review & editing. FC: Formal analysis, Writing – review & editing. HZ: Funding acquisition, Writing – review & editing.
